# Influence of gender on the vestibular evoked myogenic potential

**DOI:** 10.1590/S1808-86942011000200015

**Published:** 2015-10-19

**Authors:** Aline Tenório Lins Carnaúba, Vanessa Vieira Farias, Nastassia Santos, Aline Cabral de Oliveira, Renato Glauco de Souza Rodrigues, Pedro de Lemos Menezes

**Affiliations:** 1 Speech therapist; 2 Speech therapist; 3 Undergraduate speech therapy student; 4 Doctoral degree in otorhinolaryngology, USP. Professor at the Sergipe Federal University; 5 Doctoral degree in physics applied to medicine and biology, USP. Professor at the Alagoas State University, UNCISAL; 6 Doctoral degree in physics applied to medicine and biology, USP. Coordinator for human resources training in C&T, Alagoas State Research Foundation (Fundação de Amparo a Pesquisa do Estado de Alagoas or FAPEAL). Acoustics and Instrumentation Lab (Laboratório de Instrumentação e Acústica or LIA), Alagoas Health Science State University, UNCISAL

**Keywords:** evoked potentials, auditory, evoked potentials, motor, muscle tonus, vestibule, labyrinth

## Abstract

There is no consensus on the relevance of factors that influence gender differences in the behavior of muscles. Some studies have reported a relationship between muscle tension and amplitude of the vestibular evoked myogenic potential; others, that results depend on which muscles are studied or on how much load is applied.

**Aims:**

This study aims to compare vestibular evoked myogenic potential parameters between genders in young individuals.

**Methods:**

Eighty young adults were selected - 40 men and 40 women. Stimuli were averaged tonebursts at 500 Hz, 90 dBHL intensity, and a 10-1000 Hz bandpass filter with amplification of 10-25 microvolts per division. The recordings were made in 80 ms windows.

**Study type:**

An experimental and prospective study.

**Results:**

No significant gender differences were found in wave latency - *p* = 0.19 and *p* = 0.50 for waves P13 and N23, respectively. No differences were found in amplitude values - *p* = 0.28 *p* = 0.40 for waves P13 and N23, respectively.

**Conclusion:**

There were no gender differences in latency and amplitude factors; the sternocleidomastoid muscle strain was monitored during the examination.

## INTRODUCTION

The vestibular evoked myogenic potential (VEMP) is a reflex change in muscle activity - the vestibular-cervical reflex - which stabilizes the head when an unexpected translation occurs.^1^

The waves in this potential are defined as follows: a) latency: time elapsed between acoustic stimulation and the beginning of the most negative or positive wave value; b) wave morphology; c) peak to peak amplitude or the difference of most negative and most positive values of a wave.^2^

According to the literature, certain individual aspects should be taken into account in the VEMP test, such as the activity of the sternocleidomastoid muscle, the stimulus intensity, age, and gender.^3^

Monitoring the contraction state of the sternocleidomastoid muscle is an important parameter for adequately interpreting the VEMP test; the electromyographic level of this muscle should remain high throughout the recording.^4^, ^5^ Besides the activity of the sternocleidomastoid muscle, stimulus intensity affects the amplitude of the potential; the latency, however, is not affected and remains constant.^4^

The amplitude of the potential reflects the magnitude of the muscle reflex.^4^ This response exhibits significant interpersonal variation because of variations in muscle tone and mass, especially in males.^6^, ^7^

There are no published papers about the influence of gender on the VEMP test without the effect of musculature on responses, in other words responses due only to the function of the vestibular system, and more specifically on the role of the saccule. Therefore, the purpose of this study was to compare VEMP test parameters between genders in young adults, monitoring the state of muscle contraction to keep muscle tone similar in all the sample subjects.

## MATERIAL E METHODS

The institutional review board approved this study (Document no. 698). All participants signed a free informed consent form before participating.

Adult of both sexes with auditory thresholds equal to or below 20 dBHL (ANSI -1969) and frequency differences between ears of not more than 10 dB were enrolled.

Subjects were excluded because of: general exposure to occupational noise or to noise during leisure; a history of ear surgery; more than three ear infections in the current year; use of ototoxic drugs; the presence of systemic conditions that might worsen cochlea-vestibular diseases (diabetes, arterial hypertension, etc.); altered hormone levels; the presence of tinnitus, vertigo, dizziness or other cochlea-vestibular diseases.

There were 80 young individuals that underwent VEMP testing (40 male and 40 female).

A questionnaire was applied initially to screen the participants. Next, the following procedures were carried out: otoscopy, pure tone audiometry, and the VEMP test.

The equipment that was used for VEMP was used for VEMP testing was developed in two Brazilian public universities. The sound stimuli were presented using inserts, in an acoustic booth.

VEMP test recordings were taken with electrodes placed on the skin. The active electrode was placed on the upper half of the sternocleidomastoid muscle ipsilateral to the stimulus; the reference electrode was placed over the anterior border of the ipsilateral clavicle; the ground electrode was placed on the frontal midline.

For VEMP test recordings in the sternocleidomastoid muscle, patients were seated with the head rotated maximally to the contralateral side of the sound stimulus. The initial stimulus was right afferent followed by left afferent. The responses were replicated - recorded twice on the right and twice on the left.

The promediated stimuli were 90 dBHL tone bursts at 500 Hz, using a 10 to 1000 Hz band pass filter, amplified 10 to 25 microvolts per division. Recording were taken in 80 ms windows.

### Statistics

Data were tabulated and processed using the software Statistical Package for Social Sciences (SPSS) version 16.0. The means, standard deviations and percentiles were presented graphically on tables. The Kolmogorov-Smirnov test was applied to evaluate normalcy.

After characterizing the data with descriptive statistical techniques, the Mann-Whitney non-parametric test was applied to compare the variables. Values were significant when p was below 0.05 (*p*<0.05). The admitted beta error value was 0.1.

## RESULTS

The Mann-Whitney test with 5% significance (*p* = 0.05) was applied to analyze the results. It refers to absolute P13 and N23 latencies and amplitudes and the asymmetry index of the amplitude. The VEMP response variables were compared in terms of the evaluated side and the gender.

Confronting the right and left sides showed that there were no statistically significant differences in P13 and N23 latency and amplitude results (Tables 1 and 2).

**Table 1:** Comparison of P13 and N23 latencies (ms) in the right and left ears, in both sexes.

		*p*13 Latency			n23 Latency		
		Mean	Standard deviation	*p* value	Mean	Standard deviation	*p* value
Female	RE	14.13	1.39	0,74	24.04	1.72	0,63
	LE	14.14	1.42		24.14	1.84	
Male	RE	14.15	1.21	0,48	24.26	2.09	0,91
	LE	14.35	1.41		24.28	2.24

**Table 2:** Comparison of P13-N23 wave amplitudes (*μ*V) in the right and left ears, in both sexes.

		*p*13 Amplitude		n23 Amplitude			
		Mean	Standard deviation	*p* value	Mean	Standard deviation	*p* value
Female	RE	26.56	13.67	0,18	32.99	17.03	0,64
	LE	22.85	11.49		31.32	18.07	
Male	RE	28.29	12.14	0,17	36.89	18.26	0,14
	LE	23.36	11.51		30.38	17.19	

A gender comparison revealed no statistically significant differences in wave p13 e n23 latencies and amplitudes (Table 3). The asymmetry index value for the amplitude ranged from 26.56% to 32.98%.

**Table 3:** Comparison of P13 and N23 latencies (ms) and P13- N23 wave amplitude (*μ*V) between sexes.

	*p*13 Latency	*p*13 Amplitude	n23 Latency	n23 Amplitude
Male	14.25	25.75	24.30	33.54
Female	14.15	24.50	24.10	32.00
*p* value	0.19	0.28	0.51	0.41

## DISCUSSION

Several acoustic stimulus frequencies have been described. Our choice for a 500 Hz stimulus is because of superior responses at this and lower frequencies.^4^, ^8^, ^9^ The most frequent technique was to place the electrodes on the sternocleidomastoid muscle; authors have suggested that this yields more consistent and homogeneous responses.^2^, ^9^, ^10^ The best specific site of the electrode in the sternocleidomastoid muscle for response recordings in VEMP testing is, as research has shown, the middle third of this muscle.^11^, ^12^, ^13^, ^14^, ^15^ Other studies have shown that the best head position for activating the sternocleidomastoid muscle is maximal lateral rotation with subjects in the sitting position.^16^, ^17^, ^18^

Analysis of VEMP test responses in our sample of young adults yielded similar latency and amplitude results to those in other studies.^2^, ^4^, ^9^

A comparison of right and left sides revealed no statistically significant difference in P13 and N23 latency and amplitude results; this concurs with several studies^2^, ^12^, ^19^ that have reached similar conclusions.

There were no gender-related VEMP test alterations in P13 and N23 latency and amplitude results, or any interaural latency difference between both components, which again concurs with published results.^2^, ^20^

Amplitude responses may be affected by the level of muscle activity.^21^ This may explain why there were no gender differences in values; the activity of the sternocleidomastoid muscle was monitored throughout testing to eliminate differences and to truly evaluate only the function of the saccule. Our results were similar to those in some studies,^22^ and differed from those in others.^9^

## CONCLUSION

There were no gender differences in latency and amplitude when monitoring the tone of the sternocleidomastoid muscle during the test.
